# Lactate/albumin ratio predicts 90-day mortality of cardiogenic shock patients

**DOI:** 10.1515/med-2025-1355

**Published:** 2026-02-20

**Authors:** Haimin Liu, Junjie Zhou, Manqi Liu, Junjun Liu, Dandong Luo, Chongjian Zhang

**Affiliations:** Department of Intensive Care Unit, Heyuan People’s Hospital, Guangdong Provincial People’s Hospital Heyuan Hospital, Guangdong Provincial People’s Hospital, Heyuan, China; Department of Cardiac Surgery Intensive Care Unit, Guangdong Provincial People’s Hospital, Guangdong Academy of Medical Sciences, Southern Medical University Guangzhou, China

**Keywords:** lactate/albumin ratio, lactate, albumin, mortality, cardiogenic shock

## Abstract

**Objectives:**

Serum lactate and albumin levels upon admission are independent risk factors for poor prognosis in patients with cardiogenic shock (CS). The association between the serum lactate/albumin ratio (LAR) and mortality in patients with cardiogenic shock remains unclear. This study aims to explore the relationship between LAR at admission and the 90-day mortality of patients with CS.

**Methods:**

We performed a secondary analysis of previously published data on cardiogenic shock patients. Based on the curve, an LAR of >0.094 indicated a harmful threshold. The outcomes of cardiogenic shock Patients dichotomized according to the LAR cut-off value. Univariate logistic regression models, the receiver operating characteristic (ROC) curve in the multivariate analysis, a restricted cubic spline plot derived from multivariate logistic regression, Decision curve analysis and subgroup analyses were employed to investigate the association between LAR and mortality of cardiogenic shock.

**Results:**

A total of 176 patients were included. Univariate logistic analyses revealed a positive association between LAR and 90-day mortality of cardiogenic shock patients (HR 4.50, 95 % CI=2.64–7.66, p<0.001). The Area Under the Curve (AUC) value for LAR was 0.781 (95 % CI: 0.713–0.848), which was higher than that for initial lactate (AUC=0.768) and albumin (AUC=0.652) alone. It was not inferior even when compared to IABP-SHOCK II score (AUC=0.719). The restricted cubic spline analysis demonstrated a linear relationship between baseline LAR and the mortality of patients with cardiogenic shock. The Kaplan-Meier curves indicate lower survival rates in patients with LAR values >0.094. Decision curve analysis shows that LAR model has the best utility in intermediate-risk clinical decisions (30–75 % threshold). The final subgroup analysis showed no significant interaction of LAR with each subgroup (P for interaction: 0.057–0.948).

**Conclusions:**

The baseline Lactic/albumin Ratio (LAR) in patients experiencing cardiogenic shock shows a positive correlation with mortality associated with this condition. The findings indicate a significant increase in the mortality rate when the LAR exceeds a certain threshold. Furthermore, the LAR serves as an independent risk factor for poor prognosis in patients suffering from cardiogenic shock, with superior prognostic performance than initial lactate or serum albumin alone. Clinicians use LAR for personalized risk assessment when they encounter a medium probability scenario for 90-day mortality.

## Introduction

Cardiogenic shock (CS) is a complex hemodynamic complex syndrome, due to cardiac output, hypotension and systemic perfusion, causing systemic microcirculation dysfunction, resulting in ischemia, hypoxia, inflammation, vasoconstriction and volume overload of pathophysiological process, there is insufficient perfusion, increased serum creatinine, metabolic acidosis and elevated serum lactate biochemical performance, reflects the tissue hypoxia and cell metabolism changes, eventually leading to multiple organ system failure and death [1]. Several underlying etiologies contribute to this condition, with acute myocardial infarction being the most common. Other, less prevalent causes include new subtypes of cardiogenic shock (CS), such as fulminant myocarditis, right ventricular failure, Takotsubo syndrome, postpartum cardiomyopathy, end-stage heart valve disease, and acute decompensation of various cardiomyopathies [2], [3], [4]. Although patients with cardiogenic shock receive etiologic therapy, including mechanical circulatory supportive therapy, hospital mortality in cardiogenic shock ranges between 30 and 60 %, with nearly half of hospital deaths occurring within the first 24 h of onset, 50 % at 30 days after diagnosis, nearly 50 % within 90 days, and 58 % within one year, depending on the underlying etiology [5]. Recently, serum glucose, lactate, and creatinine have been identified as valuable biomarkers for the risk stratification of cardiogenic shock.

The importance of lactate in intensive care and cardiogenic shock has gained recognition due to its diagnostic and prognostic significance. Elevated lactate levels in these cases are attributed to either type A or type B lactic acidosis [6]. Type A lactic acidosis is prevalent in cases of cardiogenic shock, characterized by clinical evidence of tissue hypoxia. This condition arises due to insufficient cardiac output to meet the body’s oxygen demands, leading to increased anaerobic glycolysis. In contrast, Type B lactic acidosis lacks clinical evidence of tissue hypoxia and can result from various factors, including liver dysfunction, which impairs lactate clearance, as well as the use of certain medications that interfere with lactate metabolism. Additionally, basal metabolic disorders may contribute to Type B lactic acidosis by altering the mechanisms involved in lactate production and clearance [7], 8]. Lactate serves as an independent predictor of short-term mortality in cases of cardiogenic shock [4], 9]. However, the single use of lactate level prediction does not ensure a credible consequence.

Albumin, the most abundant plasma protein, functions as the primary transporter in the bloodstream. Serum albumin levels may decrease in both acute and chronic diseases due to factors such as increased capillary leakage, impaired synthesis related to liver dysfunction, malnutrition, or inflammation, thus limiting its use as a single prognostic indicator. Notably, the liver plays a crucial role in lactic acid metabolism; when liver function is compromised, it adversely affects lactic acid metabolism, leading to its accumulation. Since albumin is synthesized by the liver, its levels can reflect liver function status. Elevated lactate levels or diminished albumin levels serve as direct and indirect indicators of dysfunction in lactate metabolism, respectively [10]. Cardiogenic shock can lead to insufficient liver perfusion, inhibit the synthesis function of hepatocytes, resulting in reduced albumin synthesis. In addition, cardiogenic shock can lead to increased vascular permeability, albumin may leak from blood vessels into the tissue space, further aggravating hypoproteinemia. Studies have demonstrated that cardiogenic shock patients with albumin levels <30.0 g/L are associated with an increased risk of 30-day all-cause mortality [11]. However, albumin concentration is influenced by various factors, including nutritional status, inflammation and nephrotic syndrome. Therefore, predictions based solely on albumin concentrations may have limitations. This highlights the advantage of using LAR, which helps to reduce the confounding effects of nutritional status, inflammation and nephrotic syndrome on albumin interpretation.

To date, no study has investigated the relationship between the LAR ratio and cardiogenic shock. Given that the overall risk of all-cause mortality in short-term CS patients is unpredictable and that the predictive value of blood-derived biomarkers is relatively poor, there is a need for improved risk prediction tools to identify individuals at the highest risk of poor prognosis following CS. We hypothesized that the lactate-to-albumin ratio may serve as a potential predictor of cardiogenic shock. Consequently, this study aimed to investigate the prognostic value of the LAR during hospitalization for CS, the dynamic changes in lactate and albumin levels during ICU stays, and to further explore the predictive value of the lactate-to-albumin ratio for 90-day all-cause mortality in CS patients.

## Methods

### Study population

The CardShock study (https://doi.org/10.1002/ejhf.260) is a European prospective, observational, multicentre and multinational study on cardiogenic shock. Recruitment was conducted between October 2010 and 31 December 2012. We conducted a secondary analysis of this study. This study was carried out in the intensive care units (ICUs) of emergency departments, cardiac units, and catheter laboratories across nine tertiary hospitals in eight countries, and it received approval from the local Ethical Committee. The study received Ethical Approval from various committees across Europe, including: Athens – Ethics Committee of Attikon University Hospital; Barcelona – Health Research Ethics Committee of the Hospital de Sant Pau; Brescia – Ethics Committee of the Province of Brescia; Brno – Ethics Committee of University Hospital Brno; Helsinki – The Ethics Committee, Department of Medicine, The Hospital District of Helsinki and Uusimaa; Porto – Ethics Committee of S. João Hospital Center/Porto Medical School; Rome – Ethical Committee Sant’Andrea Hospital; Warsaw – Local Bioethics Committee of the Institute of Cardiology; and Copenhagen – approved by the Danish Protection Agency. The original data from that study are publicly available for free access and analysis at the following link: https://doi.org/10.1371/journal.pone.0217006.s002. The initial study included CS patients from October 2010 to 31 December 2012.

The study enrolled consecutive patients aged over 18 years who were experiencing shock due to cardiac causes. The inclusion criteria specified a systolic blood pressure of less than 90 mmHg, accompanied by adequate filling pressures for at least 30 min, or the necessity for vasopressor therapy to maintain a systolic blood pressure greater than 90 mm Hg. Additionally, one or more signs of hypoperfusion had to be present, including altered mental status or confusion, cold extremities, oliguria of less than 0.5 mL/kg/h over the preceding 6 h, or a blood lactate level exceeding 2 mmol/L. Patients were excluded if their shock was caused by ongoing hemodynamically significant arrhythmias or if they had undergone cardiac or noncardiac surgery.

**Figure 1:**
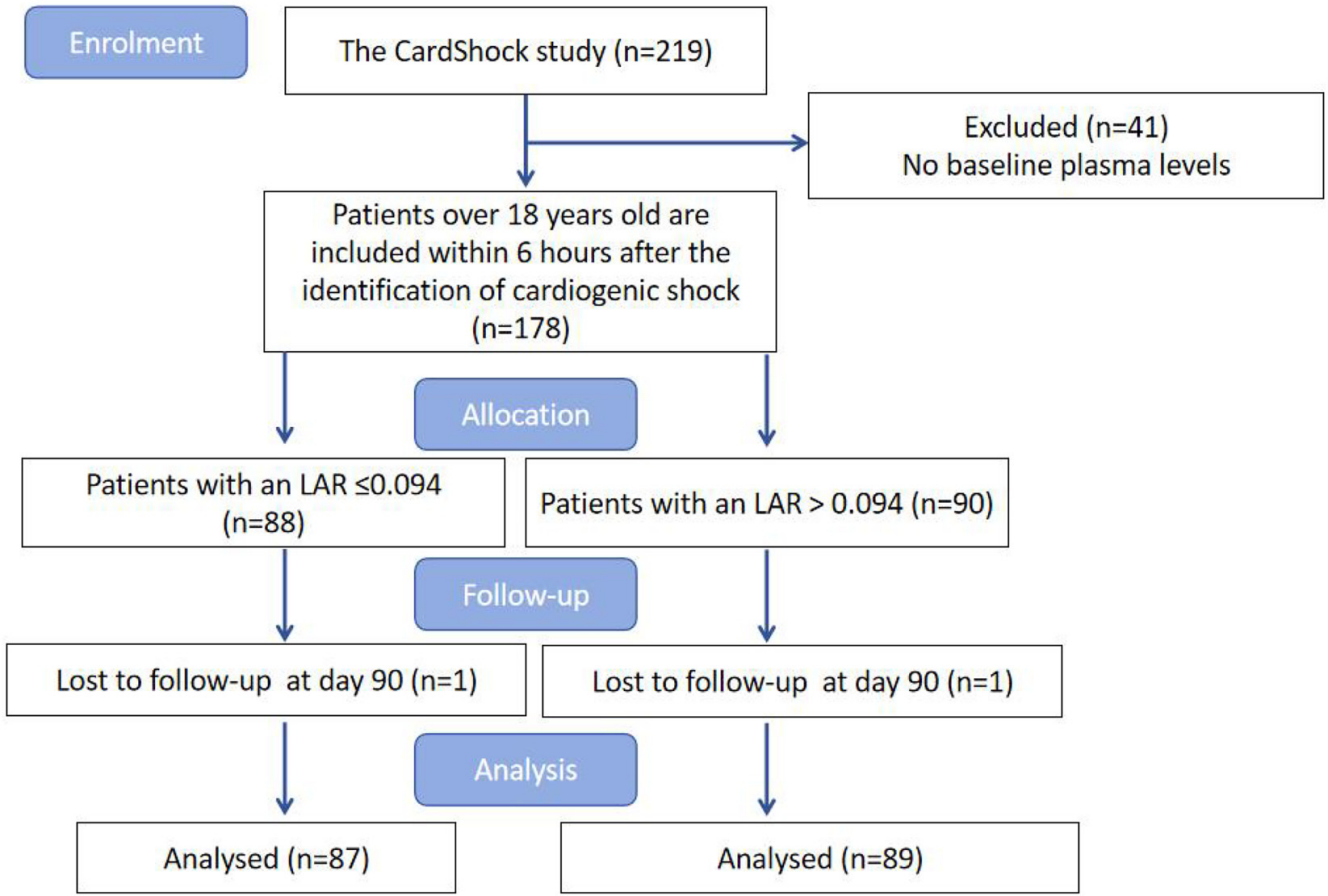
Schematic diagram of the study sample selection steps.

The screening protocol used in this study is detailed in Figure 1. The CardShock study database includes relatively comprehensive medical information on 219 cardiogenic shock patients. Forty one patient samples without baseline plasma levels were excluded. The study cohort included 178 patients with plasma samples available at baseline, forming the study population. The primary endpoint was 90-day all-cause mortality. Participants’ health status during follow-up was confirmed through direct contact with patients or their families, or by reviewing population and hospital registers. Two patients were lost to follow-up during the study. The protocol was approved by the Ethics Review Committee of Guangdong Provincial People’s Hospital (code: KY2024-1064-01) and conducted in accordance with the Declaration of Helsinki. Informed consent to participate was obtained from all participants or their legal representatives.

### Data collection

The study collected comprehensive demographic data, including age, sex, body mass index, smoking status (current smoker and ex-smoker), and medical history, which encompassed coronary artery disease, myocardial infarction, coronary artery bypass grafting, heart failure with reduced ejection fraction, and diabetes mellitus. Additionally, we documented medications administered at admission, such as angiotensin-converting enzyme inhibitors, angiotensin receptor blockers, calcium-channel blockers, and beta-blockers. Clinical presentations were assessed, including confusion, oliguria, acute coronary syndrome etiology, lung edema observed on X-ray, systolic blood pressure, diastolic blood pressure, mean arterial pressure, left ventricular ejection fraction, and estimated glomerular filtration rate. Researchers calculated the CardShock risk score and IABP-SHOCK II score. Baseline laboratory test results were obtained, including hemoglobin, leukocytes, N-terminal prohormone of B-type natriuretic peptide, C-reactive protein, alanine aminotransferase, lactate, albumin, and alkaline phosphatase. Complications from percutaneous coronary intervention were noted, alongside the time from shock detection to baseline assessment and the vital status of patients at 90 days.

### Statistical analysis

The Kolmogorov–Smirnov test was employed to assess normality. Continuous variables that adhered to a normal distribution were presented as mean ± standard deviation, while those that did not conform to normality were represented as M (Q1, Q3). Categorical data were expressed in terms of frequency (%). Group differences were analyzed accordingly.

Fisher’s exact test and Chi-squared tests were employed for the analysis of dichotomous variables. To identify risk factors influencing 90-day mortality in patients with cardiogenic shock, univariate analyses were performed. Subsequently, the statistically significant variables identified in the univariate analysis were further analyzed using the receiver operating characteristic (ROC) curve in the multivariate analysis. The unadjusted survival curves were then plotted by the Kaplan-Meier (KM) method, and the two groups of curves were compared using the log Rank test. We then utilized logistic regression to establish the relationship between various variables and 90-day mortality rates. The basis for covariate selection is as follows: First, perform collinearity analysis and eliminate the covariates with VIF >5 (Supplementary appendix 1). Second, based on p<0.1 method and effect value change method. We constructed and employed five models to adjust for confounding factors. The selection of confounding factors in the multivariate regression model was based on the results of the univariate regression and clinical expertise. The potential mediating roles of demographics, medical history, medications at admission, clinical presentation, and baseline laboratory test results on the association between the lactate dehydrogenase to albumin ratio and mortality were evaluated based on change-in-estimate and associated p-values. Model 1 was unadjusted. In the univariate regression analysis, the three demographic variables sex, age and BMI were included in Model 2. The model 3 was screened for variables using the change-in-estimate method, adjusting for two variables with a 10 % change in effect value: LEVF (left ventricular ejection fraction) and eGFR (estimated glomerular filtration rate) [12]. Model 4 adjusts p<0.1 for 11 variables, including age, LVEF, eGFR, current smoker, ex-smoker, confusion, oliguria, coronary artery disease, PCI complications, mean arterial pressure, alkaline phosphatase. Model 5 adjusts the 13 variables of models 2, 3, and Model 4, including sex, age, BMI, LVEF, eGFR, Current smoker, ex-smoker, confusion, oliguria, coronary artery disease, PCI complications, mean arterial pressure, alkaline phosphatase. We illustrated the dose-effect relationship between LAR and the 90-day mortality rate through a restricted cubic spline plot derived from multivariate logistic regression. Decision curve analysis (DCA) was conducted by ggscidca. Finally, we conducted a subgroup analysis to investigate whether LAR had any impact on different subgroups, including gender, age, confusion, oliguria, medical history, which encompassed coronary artery disease, myocardial infarction, coronary artery bypass grafting, heart failure with reduced ejection fraction, and diabetes mellitus, current smoker, ex-smoker, lung edema observed on X-ray, and complications related to Percutaneous Coronary Intervention. All analyses were performed using R software. p<0.05 was considered statistically significant.

## Results

### Baseline data of included patients

A total of 176 patients with complete lactate values and albumin records, as shown in Table 1. In addition, 90-day mortality was significantly higher in cardiogenic shock patients with an LAR >0.094 than in those with LAR ≤0.094 [56 (62.9 %) vs. 18 (20.7 %), p<0.001]. As shown in Table 1, patients with a high LAR were older (p<0.05). Individuals with a high LAR had a higher prevalence of current smoking (p<0.01), a greater history of diabetes mellitus (p<0.01), lower left ventricular ejection fraction (LVEF) (p<0.01), lower estimated glomerular filtration rate (eGFR) (p<0.001), higher CardShock risk scores (p<0.001), higher IABP-SHOCK II score (p<0.001), elevated alanine aminotransferase (ALT) levels (p<0.001), increased lactate levels (p<0.001), and lower albumin (ALB) levels (p<0.001).

**Table 1:** Baseline data of cardiogenic shock Patients dichotomized according to the Lactate/albumin ratio cut-off value.

	Low LAR (n=87)	High LAR (n=89)	p-Value
**Characteristics**			
Age, years	64.0 [55.7; 74.3]	68.6 [61.4; 77.0]	0.031
Male, n, %	68 (78.2 %)	62 (69.7 %)	0.266
BMI, kg/m2; mean, SD	26.6 ± 3.9	27.2 ± 4.3	0.349
Current smoker	44 (50.6 %)	26 (29.2 %)	0.006
Ex-smoker	15 (17.2 %)	20 (22.5 %)	0.496
**Medical history**			
Coronary artery disease	26 (29.9 %)	32 (36.0 %)	0.486
Previous MI	21 (24.1 %)	24 (27.0 %)	0.797
Prior CABG	4 (4.6 %)	7 (7.9 %)	0.559
Previous MI or CABG	21 (24.1 %)	25 (28.1 %)	0.671
History of HFrEF	7 (8.0 %)	15 (16.9 %)	0.124
Diabetes mellitus	16 (18.4 %)	36 (40.4 %)	0.002
**Medications in use at admission**			
ACEI	21 (24.1 %)	31 (34.8 %)	0.165
ARB	16 (18.4 %)	10 (11.2 %)	0.261
Calcium-channel blockers	12 (13.8 %)	11 (12.4 %)	0.869
Beta-blocker	32 (36.8 %)	36 (40.4 %)	0.953
**Clinical presentation**			
Confusion	48 (55.2 %)	67 (75.3 %)	0.008
Oliguria (<0.5 mL/kg/h over 6 h)	33 (37.9 %)	61 (68.5 %)	<0.001
ACS etiology	71 (81.6 %)	70 (78.7 %)	0.762
Lung oedema on X-ray	28 (32.2 %)	31 (34.8 %)	0.832
Systolic BP, mmHg; median (IQR)	80.0 [70.0; 85.0]	77.0 [69.0; 81.0]	0.143
Diastolic BP, mmHg	45.0 [40.0; 53.0]	45.0 [40.0; 55.0]	0.977
Mean arterial pressure, mmHg	57.0 [51.3; 63.0]	55.0 [50.0; 63.0]	0.479
LVEF, %	35.0 [25.0; 46.0]	30.0 [20.0; 40.0]	0.008
eGFR	70.9 [55.3; 97.6]	45.5 [33.5; 70.4]	<0.001
**CardShock risk score**			<0.001
0	4 (4.6 %)	0 (0.0 %)	
1	8 (9.2 %)	0 (0.0 %)	
2	14 (16.1 %)	3 (3.4 %)	
3	25 (28.7 %)	9 (10.1 %)	
4	15 (17.2 %)	10 (11.2 %)	
5	15 (17.2 %)	28 (31.5 %)	
6	4 (4.6 %)	20 (22.5 %)	
7	2 (2.3 %)	12 (13.5 %)	
8	0 (0.0 %)	6 (6.7 %)	
9	0 (0.0 %)	1 (1.1 %)	
	3.0 [2.0; 4.0]	5.0 [4.5; 6.0]	<0.001
**IABP-SHOCK II score**	1.0 [1.0; 2.0]	3.0 [2.0; 5.0]	<0.001
**Laboratory test results at baseline**			
Haemoglobin	131.1 ± 23.9	126.2 ± 22.3	0.163
Leucocytes	12.7 [9.8; 16.4]	14.0 [10.1; 18.0]	0.176
NT-proBNP	1,860.0 [456.0; 6,846.5]	4,838.0 [1,078.0; 12,294.0]	0.131
CRP	8.0 [4.0; 50.5]	18.5 [4.5; 59.9]	0.486
ALT	26.1 [16.6; 53.0]	72.2 [35.5; 133.0]	<0.001
Lactate	1.7 [1.2; 2.2]	5.6 [3.6; 8.2]	<0.001
ALB	31.8 ± 5.5	27.2 ± 6.4	<0.001
ALP	63.0 [50.4; 78.1]	59.7 [48.2; 84.3]	0.815
LAR	0.1 [0.0; 0.1]	0.2 [0.1; 0.3]	<0.001
**Angiographic findings**			
PCI complications	20 (23.0 %)	23 (25.8 %)	0.791
Time from detection of shock to baseline; min; median (IQR)	90.0 [0.0; 240.0]	120.0 [30.0; 210.0]	0.592
Vital status at 90 days	18 (20.7 %)	56 (62.9 %)	<0.001

Results shown as n (%) for categorical and mean (SD) or median (IQR) for continuous variables. BMI, body mass index; CABG, coronary artery bypass grafting; MI, myocardial infarction; HFrEF, heart failure with reduced ejection fraction; ACEI, angiotensin-converting enzyme inhibitor; ARB, angiotensin receptor blocker; ACS, acute coronary syndrome; BP, blood pressure; LVEF, left ventricular ejection fraction; eGFR, estimated glomerular filtration rate; NT-proBNP, N-terminal prohormone of B-type natriuretic peptide; CRP, C-reactive protein; ALT, alanine aminotransferase; ALB, albumin; ALP, alkaline phosphatase; LAR, lactate/albumin ratio; PCI, percutaneous coronary intervention; SD, standard deviation; IQR, interquartile range.

### Univariate logistic analyses of risk factors associated with death during the 90 days of follow-up

To identify the risk factors affecting 90-day mortality of cardiogenic shock patients, univariate analyses were conducted (Table 2). Risk factors associated with high 90-day mortality of cardiogenic shock Patients were age (p<0.001, HR 1.04, 95 % CI 1.02–1.06), medical history of coronary artery disease (p=0.001, HR 2.18, 95 % CI 1.38–3.45), previous medical history of MI (p<0.01, HR 2.18, 95 % CI 1.36–3.49), previous medical history of CABG (p<0.01, HR 2.92, 95 % CI 1.49–5.70), history of diabetes mellitus (p<0.01, HR 2.08, 95 % CI 1.31–3.30), confusion (p<0.001, HR 2.81, 95 % CI 1.57–5.04), oliguria (p<0.001, HR 2.87, 95 % CI 1.73–4.76), higher LAR (p<0.001, HR 4.50, 95 % CI 2.64–7.66).

**Table 2:** Univariate logistic analyses of risk factors associated with death during the 90 days of follow-up.

Variables	Univariate analysis	
	HR (95 % CI)	p-Value
Age, years	1.04 (1.02–1.06)	<0.001
Male gender	0.67 (0.41–1.10)	0.12
BMI	1.00 (0.94–1.06)	0.98
Current smoker	0.60 (0.37–0.98)	0.037
Ex-smoker	0.93 (0.52–1.67)	0.81
**Medical history**		
Coronary artery disease	2.18 (1.38–3.45)	0.001
Previous MI	2.18 (1.36–3.49)	0.002
Prior CABG	2.92 (1.49–5.70)	0.006
History of HFrEF	1.76 (0.96–3.20)	0.084
Diabetes mellitus	2.08 (1.31–3.30)	0.003
**Medications in use at admission**		
ACEI	1.08 (0.65–1.77)	0.77
ARB	1.11 (0.60–2.07)	0.73
Calcium-channel blockers	0.94 (0.48–1.83)	0.85
Beta-blocker	1.42 (0.90–2.24)	0.14
**Clinical presentation**		
Confusion	2.81 (1.57–5.04)	<0.001
Oliguria (<0.5 mL/kg/h over 6 h)	2.87 (1.73–4.76)	<0.001
ACS etiology	1.75 (0.90–3.41)	0.079
Lung oedema on X-ray	1.29 (0.81–2.06)	0.29
Mean arterial pressure	0.97 (0.94–0.99)	0.003
LVEF	0.97 (0.95–0.99)	<0.001
eGFR	0.98 (0.97–0.99)	<0.001
**Laboratory test results at baseline**		
Haemoglobin	0.99 (0.98–1.00)	0.031
Leucocytes	1.02 (0.98–1.06)	0.43
NT-proBNP	1.00 (1.00–1.00)	<0.001
CRP	1.00 (1.00–1.00)	0.64
ALT	1.00 (1.00–1.00)	0.76
ALP	1.00 (0.99–1.01)	0.77
LAR dichotomous		
Low	1	
High	4.50 (2.64–7.66)	<0.001
**Angiographic findings**		
PCI complications	1.59 (0.97–2.60)	0.074
Time from detection of shock to baseline	1.00 (1.00–1.00)	0.094

HR, hazard ratiointerval; CI, confidence; BMI, body mass index; CABG, coronary artery bypass grafting; MI, myocardial infarction; HFrEF, heart failure with reduced ejection fraction; ACEI, angiotensin-converting enzyme inhibitor; ARB, angiotensin receptor blocker; ACS, acute coronary syndrome; BP, blood pressure; LVEF, left ventricular ejection fraction; eGFR, estimated glomerular filtration rate; NT-proBNP, N-terminal prohormone of B-type natriuretic peptide; CRP, C-reactive protein; ALT, alanine aminotransferase; ALP, alkaline phosphatase; LAR, lactate/albumin ratio; PCI, percutaneous coronary intervention; SD, standard deviation; IQR, interquartile range.

### The evaluation of potentially useful markers for 90-day mortality following discharge

**Figure 2:**
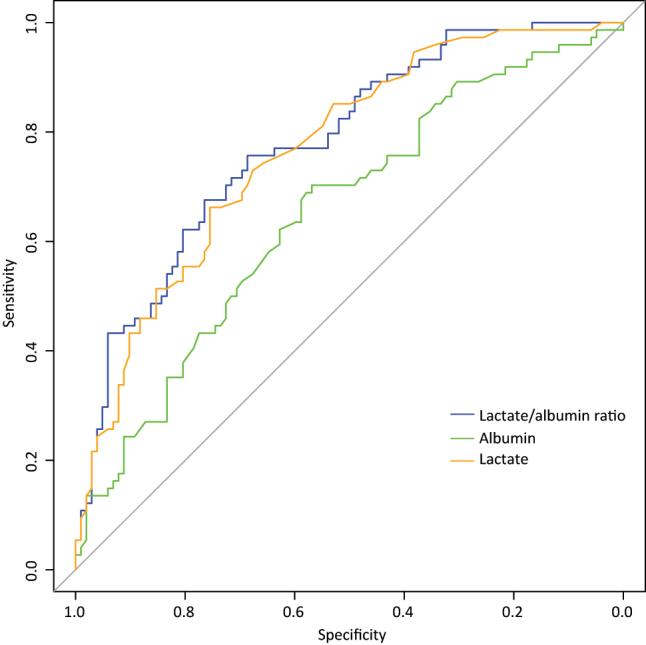
Receiver operating characteristic curve analyses of the lactate, albumin, and LAR values to predict postoperative 90-day mortality in cardiogenic shock patients.

**Figure 3:**
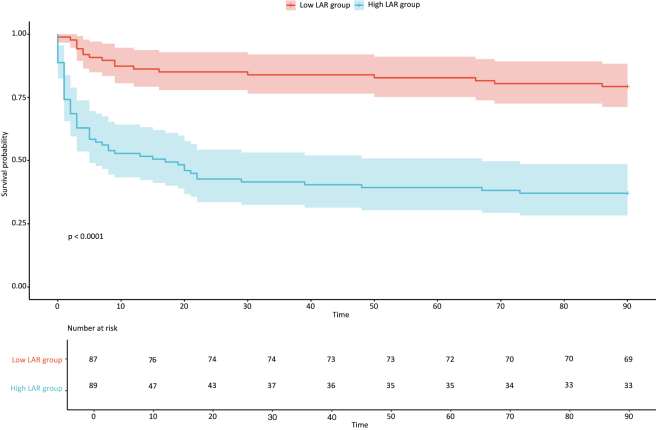
Kaplan-Meier curves illustrate the 90-day survival rates of patients experiencing cardiogenic shock following their discharge from the intensive care unit.

The evaluation of potentially useful markers for 90-day mortality following discharge is crucial for improving patient outcomes. The cutoff values, area under the curve (AUC), sensitivity, specificity, positive and negative predictive values, diagnostic odds ratio, and accuracy of lactate, albumin, and the LAR at admission are summarized in Table 3 as predictors of mortality within 90 days post-ICU discharge. The optimal cut-off values were determined to be 3.250 mmol/L for lactate within 3 h of identifying cardiogenic shock, 30.650 g/L for albumin, and 0.094 mmol/g for the LAR (Table 3). Among these markers, LAR demonstrated the highest AUC and sensitivity. Specifically, the LAR (AUC=0.781, 95 % CI=0.713–0.848) measured within 3 h of identifying cardiogenic shock exhibited a greater AUC than lactate (AUC=0.768, 95 % CI=0.699–0.837) and albumin alone (AUC=0.652, 95 % CI=0.570–0.734) (Figure 2).It demonstrated that the LAR had a higher predictive value than lactate and albumin for 90-day mortality in patients with cardiogenic shock. The 90-day survival rates are illustrated in Figure 3, which indicates lower survival in patients with LAR>0.094 mmol/g.

**Table 3:** The evaluation of potentially useful markers for 90-day mortality following discharge.

Predictor	AUC (95 % CI)	Cut-off value	Sensitivity % (95 % CI)	Specificity % (95 % CI)	PPV % (95 % CI)	NPV (95 % CI)	p-Value
Lactate	0.768(0.699–0.837)	3.250	66.2(55.4–77.0)	75.5(67.1–83.8)	66.2(55.4–77.0)	75.5(67.1–83.8)	<0.001
ALB	0.652(0.570–0.734)	30.650	70.3(59.9–80.7)	56.9(47.3–66.5)	54.2(44.2–64.1)	72.5(62.7–82.3)	<0.001
LAR	0.781(0.713–0.848)	0.094	75.7(65.9–85.5)	68.6(59.6–77.6)	63.6(53.6–73.7)	79.5(71.1–88.0)	<0.001

AUC, area under curve; CI, confidence internal; ALB, albumin; PPV, positive predictive value; NPV, negative predictive value; LAR, lactate/albumin ratio.

### Comparison of cardiogenic shock risk score models

**Figure 4:**
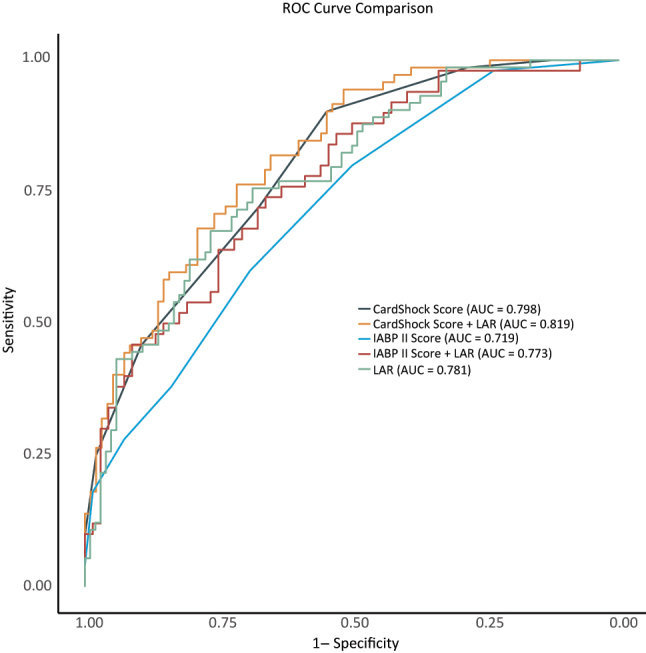
Diagnostic accuracy of CardShock Score, CardShock Score + LAR, IABP II Score, IABP II Score + LAR and LAR in mortality prediction.

The cutoff values, area under the curve (AUC), sensitivity, specificity, positive and negative predictive values, diagnostic odds ratio, and accuracy of IABP-SHOCK II score, IABP-SHOCK II score + LAR CardShock risk score, CardShock risk score + LAR and the LAR at admission are summarized in Table 4 and Figure 4 as cardiogenic shock risk score models. The optimal cut-off values were determined to be 1.500 for IABP-SHOCK II score, 0.344 for IABP-SHOCK II score + LAR, 3.500 for CardShock risk score, 0.445 for CardShock risk score + LAR and 0.094 mmol/g for the LAR (Table 4). Addition of LAR improved the risk prediction model compared with the IABP-SHOCK II score alone (AUC=0.773, 95 % CI=0.689–0.857, p=0.019 for comparison of nested models). Which indicates that LAR has incremental value for IABP-SHOCK II score. Addition of LAR did not improve the risk prediction model compared with the CardShock risk score alone (AUC=0.819, 95 % CI=0.757–0.881, p=0.051 for comparison of nested models). When compared to the LAR reference group, the p-value of IABP-SHOCK II score was 0.280. Which suggests that the predictive value of LAR is equivalent to that of IABP-SHOCK II score. When compared to the LAR reference group, the p-value of CardShock risk score was 0.712. Which suggests that the predictive value of LAR is equivalent to that of CardShock risk score.

**Table 4:** Comparison of cardiogenic shock risk score models.

Model	AUC (95 % CI)	Cut-off value	Sensitivity % (95 % CI)	Specificity % (95 % CI)	PPV % (95 % CI)	NPV (95 % CI)	p-Value
IABP-SHOCK II score	0.719(0.629–0.808)	1.500	80.0(68.9–91.1)	50.0(38.1–61.9)	54.1(42.7–65.4)	77.3(64.9–89.7)	0.280
IABP-SHOCK II score + LAR	0.773(0.689–0.857)	0.344	74.0(61.8–86.2)	66.2(54.9–77.4)	61.7(49.4–74.0)	77.6(66.9–88.3)	0.019
CardShock risk score	0.798(0.734–0.862)	3.500	90.3(83.4–97.1)	54.7(44.7–64.7)	60.2(51.0–69.4)	88.1(79.9–96.4)	0.712
CardShock risk score + LAR	0.819(0.757–0.881)	0.445	76.4(66.6–86.2)	71.6(62.5–80.6)	67.1(56.9–77.2)	80.0(71.5–88.5)	0.051
LAR	0.781(0.713–0.848)	0.094	75.7(65.9–85.5)	68.6(59.6–77.6)	63.6(53.6–73.7)	79.5(71.1–88.0)	<0.001

AUC, area under curve; CI, confidence internal; PPV, positive predictive value; NPV, negative predictive value; LAR, lactate/albumin ratio.

IABP-SHOCK II score and CardShock risk score are more complex, while LAR is simpler, but has similar predictive value, which shows that LAR is better. The LAR combined with IABP-SHOCK II score showed better predictive value than the IABP-SHOCK II score alone (statistically significant). When combined with CardShock risk score, although no statistically significant difference was observed, the absolute values were higher.

### The relationship between the distribution of LAR and 90-day mortality

We developed and employed five models to identify significant correlations between the LAR and various clinical outcomes. Table 5 presents the hazard ratios (HR) and 95 % confidence intervals (CI) for these models. We utilized Cox proportional hazards analysis, using the low LAR group as the reference, to assess the association between LAR and mortality in patients with cardiogenic shock. In the Model 1, the HR for 90-day mortality for patients in the LAR per 0.01, LAR per 1 standard deviation (SD), and high LAR groups were 1.03 (HR: 1.03, 95 % CI: 1.02–1.04, p<0.001), 1.75 (HR: 1.75, 95 % CI: 1.52–2.03, p<0.001), and 4.50 (HR: 4.50, 95 % CI: 2.64–7.66, p<0.001), respectively, when compared to the low LAR reference group. After controlling for variables such as sex, age and BMI, the adjusted hazard ratios (HRs) for LAR per 0.01, LAR per 1 standard deviation (SD), and high LAR groups compared to the low LAR group were 1.03 (HR: 1.03, 95 % CI: 1.02–1.04, p<0.001), 1.82 (HR: 1.82, 95 % CI: 1.56–2.13, p<0.001), and 4.10 (HR: 4.10, 95 % CI: 2.40–7.01, p<0.01), respectively. In the Model 3, the adjusted hazard ratios (HRs) for the LAR per 0.01, LAR per 1 SD, and high LAR groups compared to low LAR were 1.02 (HR: 1.02, 95 % CI: 1.01–1.03, p<0.001),1.58 (HR: 1.58, 95 % CI: 1.34–1.86, p<0.001), and 3.04 (HR: 3.04, 95 % CI: 1.73–5.36, p<0.01), respectively. An investigation of in-hospital mortality and mortality after 90 days yielded comparable findings. In the Model 4, the adjusted HRs for the LAR per 0.01, LAR per 1 SD, and high LAR groups compared to low LAR were 1.02 (HR: 1.02, 95 % CI: 1.01–1.03, p<0.001), 1.48 (HR: 1.48, 95 % CI: 1.24–1.78, p<0.001), and 2.66 (HR: 2.66, 95 % CI: 1.46–4.83, p<0.01), respectively. In the Model 5, the adjusted HRs for the LAR per 0.01, LAR per 1 SD, and high LAR groups compared to low LAR were 1.02 (HR: 1.02, 95 % CI: 1.01–1.03, p<0.001), 1.53 (HR: 1.53, 95 % CI: 1.26–1.85, p<0.001), and 2.59 (HR: 2.59, 95 % CI: 1.43–4.69, p=0.002), respectively.

**Table 5:** Cox proportional hazard ratios (HR) for all-cause mortality.

	Model 1			Model 2			Model 3			Model 4			Model 5		
Characteristic	HR^a^	95 % CI^a^	p-Value	HR^a^	95 % CI^a^	p-Value	HR^a^	95 % CI^a^	p-Value	HR^a^	95 % CI^a^	p-Value	HR^a^	95 % CI^a^	p-Value
LAR per 0.01	1.03	1.02, 1.04	<0.001	1.03	1.02, 1.04	<0.001	1.02	1.01, 1.03	<0.001	1.02	1.01, 1.03	<0.001	1.02	1.01, 1.03	<0.001
LAR per 1 SD	1.75	1.52, 2.03	<0.001	1.82	1.56, 2.13	<0.001	1.58	1.34, 1.86	<0.001	1.48	1.24, 1.78	<0.001	1.53	1.26, 1.85	<0.001
Group LAR															
Low	1.00	–		1.00	–		1.00	–		1.00	–		1.00	–	
High	4.50	2.64, 7.66	<0.001	4.10	2.40, 7.01	<0.001	3.04	1.73, 5.36	<0.001	2.66	1.46, 4.83	0.001	2.59	1.43, 4.69	0.002

^a^HR, hazard ratio; CI, confidence interval; Model 1, unadjusted; Model 2, adjusted for sex, age and BMI; Model 3, adjusted for LEVF (left ventricular ejection fraction) and eGFR (estimated glomerular filtration rate); Model 4, adjusted for age, LVEF, eGFR, current smoker, ex-smoker, confusion, oliguria, coronary artery disease, PCI complications, mean arterial pressure, alkaline phosphatase; Model 5, sex, age, BMI, LVEF, eGFR, current smoker, ex-smoker, confusion, oliguria, coronary artery disease, PCI complications, mean arterial pressure, alkaline phosphatase.

**Figure 5:**
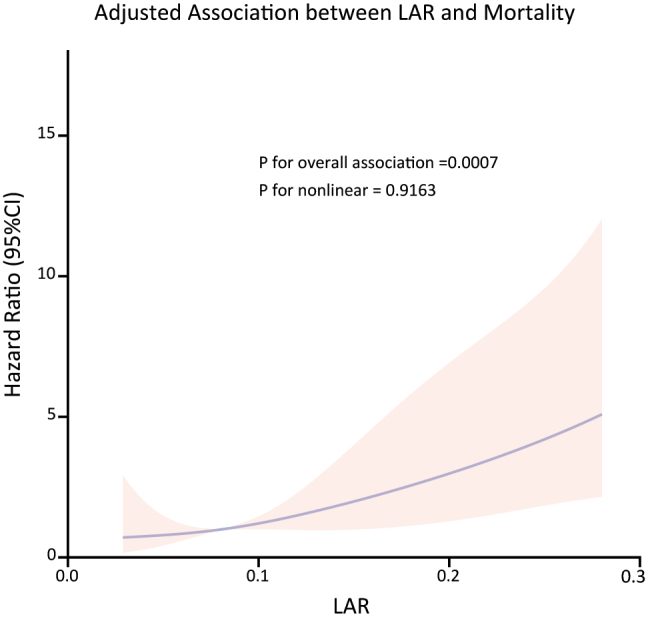
The restricted cubic spline analysis of the LAR and 90-day mortality in patients with cardiogenic shock.

After correcting for confounding variables, the restricted cubic spline analysis demonstrated a linear association between the LAR and 90-day mortality (p for linearity=0.9163, p<0.001) (Figure 5). The mortality rate among patients with cardiogenic shock increased in correlation with LAR levels.

### Decision curves corresponding to LAR, CardShock risk and IABP-SHOCK II score, respectively

**Figure 6:**
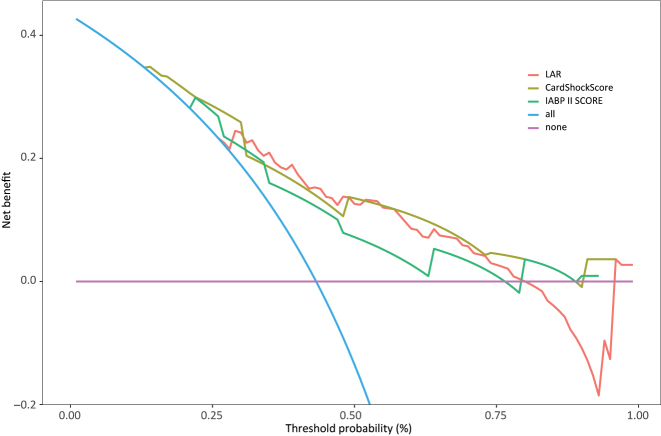
Decision curve analysis.

Decision curve analysis revealed significant threshold-dependent variation in clinical utility among the three models (Figure 6). The LAR model demonstrated superior net benefit at intermediate threshold probabilities (30–75 %), achieving peak performance at approximately 50 % probability (net benefit≈0.12). Throughout this range, LAR maintained significantly higher net benefit than both CardShock risk score and IABP-SHOCK II score. At thresholds below 30 %, CardShock risk score showed better performance, while beyond 75 %, LAR’s net benefit declined sharply into negative values whereas the other models maintained minimal positive net benefit. All models outperformed the “treat all” and “treat none” strategies within their optimal ranges.

The LAR model shows optimal utility for intermediate-risk clinical decisions (30–75 % threshold), making it particularly valuable for guiding treatment in patients where intervention risks require careful consideration against potential benefits. These findings support using LAR for personalized risk assessment when clinicians encounter moderate probability scenarios in 90-day mortality prediction.

### Sensitivity analysis and subgroup analysis of high LAR threshold

**Figure 7:**
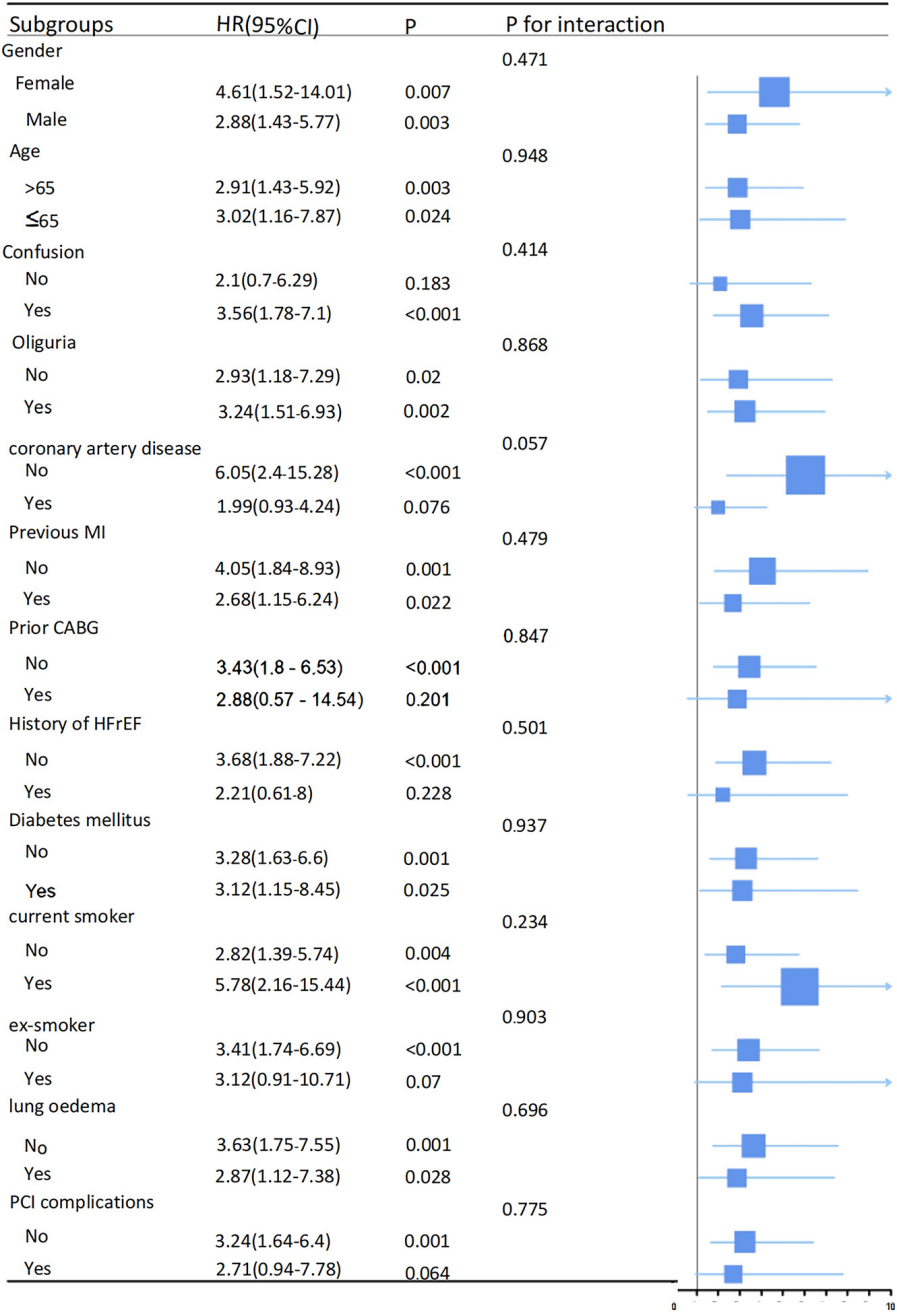
Forest plot to evaluate the association between LAR and mortality in patients with cardiogenic shock.

We conducted a subgroup analysis to evaluate the association between the LAR and mortality in patients experiencing cardiogenic shock. This analysis included variables such as gender, age, confusion, oliguria, medical history, which encompassed coronary artery disease, myocardial infarction, coronary artery bypass grafting, heart failure with reduced ejection fraction, and diabetes mellitus, current smoker, ex-smoker, lung edema observed on X-ray, and complications related to Percutaneous Coronary Intervention (Figure 7). Among females, the HR was 4.61 (95 % CI: 1.52–14.01), while for males, it was 2.88 (95 % CI: 1.43–5.77). In the subgroup of individuals aged ≤65 years, the hazard ratio (HR) was 3.02 (95 % CI: 1.16–7.87). For those aged >65 years, the HR was 2.91 (95 % CI: 1.43–5.92). Individuals presenting with confusion showed an HR of 3.56 (95 % CI: 1.78–7.10), and those with oliguria had an HR of 3.24 (95 % CI: 1.51–6.93). In contrast, individuals without oliguria had an HR of 2.93 (95 % CI: 1.18–7.29). Those without coronary artery disease had an HR of 6.05 (95 % CI: 2.40–15.28), while individuals with a history of myocardial infarction had an HR of 2.68 (95 % CI: 1.15–6.24). For those without myocardial infarction, the HR was 4.05 (95 % CI: 1.84–8.93). Patients who did not undergo coronary artery bypass grafting had an HR of 3.43 (95 % CI: 1.80–6.53), and those without heart failure with reduced ejection fraction had an HR of 3.68 (95 % CI: 1.88–7.22). Individuals presenting with diabetes mellitus showed an HR of 3.12 (95 % CI: 1.15–8.45), and those without diabetes mellitus had an HR of 3.28 (95 % CI: 1.63–6.60). Current smokers exhibited an HR of 5.78 (95 % CI: 2.16–15.44), whereas non-smokers had an HR of 2.82 (95 % CI: 1.39–5.74). Ex-smokers had an HR of 3.12 (95 % CI: 0.91–10.71), compared to non-ex-smokers, who had an HR of 3.41 (95 % CI: 1.74–6.69). Individuals with lung edema on X-ray had an HR of 2.87 (95 % CI: 1.12–7.38), while those without lung edema had an HR of 3.63 (95 % CI: 1.75–7.55). Finally, individuals without complications from percutaneous coronary intervention had an HR of 3.24 (95 % CI: 1.64–6.40). The forest plot (Figure 7) showed no significant interaction of LAR with each subgroup (p for interaction: 0.057–0.948), suggesting that LAR serves as an independent prognostic factor.

## Discussion

The primary conclusions drawn from this single-center retrospective study are as follows: (i) Baseline LAR in patients experiencing cardiogenic shock demonstrates a non-linear relationship with mortality associated with cardiogenic shock. (ii) There is a significant increase in mortality rates when the LAR is excessively elevated. The LAR is identified as an independent risk factor for poor prognosis in cardiogenic shock. (iii) LAR measured early in the course of cardiogenic shock has a greater association with mortality of cardiogenic shock than either initial lactate or initial albumin alone. To the best of our knowledge, this is the first clinical study to investigate the relationship between LAR, a novel inflammatory marker, and mortality in patients with cardiogenic shock.

Serum lactate levels are a marker of tissue hypoxia often used in the assessment of severity of illness for patients with shock and multiple-organ dysfunction syndrome (MODS). Lactate has been identified as a strong independent predictor of in-hospital mortality among critically ill patients. The prevailing paradigm posits that elevated lactate concentrations result from anaerobic glycolysis, which is triggered by tissue hypoperfusion, hypoxia, or a combination of both. In essence, heightened lactate levels signify a profound stress response occurring alongside tissue hypoperfusion, thereby establishing lactate as a biomarker for both hypoperfusion and adrenergic stress [13]. Biomarkers, particularly serum lactate, serve as crucial diagnostic tools for assessing overall health status and recognizing organ failure. Cardiogenic shock represents a systemic condition resulting from general end-organ hypoperfusion. The prognostic significance of lactate in cardiogenic shock has been validated in numerous studies conducted over the past few decades [14]. Lactic acidosis is a critical diagnostic criterion for hypoperfusion. The severity of lactic acidosis, which reflects the magnitude and duration of hypoperfusion, is a more important physiological variable [15]. This is consistent with our findings. In this study, our study population consisted of critically ill patients with cardiogenic shock. We found that with the increase of L/A ratio, lactate level increased, and the mortality of patients increased significantly. In conclusion, tissue hypoperfusion due to cardiogenic shock leads to elevated lactate levels. However, lactate has complex metabolic and clearance mechanisms, it is not easy to accurately identify a disease by using this indicator alone.

Hypoalbuminemia is prevalent among acutely ill patients, with reported rates ranging from 21% to 70 % in hospitalized individuals. Albumin is the most abundant protein in plasma and serves as a primary transporter for numerous compounds in the blood. The occurrence of hypoalbuminemia in acute illness can be attributed to increased capillary permeability or decreased synthesis, which may result from inflammation, underlying malnutrition, or hepatic synthetic dysfunction [16], 17]. Albumin has been demonstrated to be a reliable predictor of both short- and long-term outcomes across various clinical settings, including patients with acute coronary syndrome, acute heart failure, acute ischemic stroke, sepsis, chronic kidney disease, and cardiogenic shock [11], [18], [19], [20], [21], [22]. Serum albumin, while traditionally considered a marker of nutritional status, is also a negative acute-phase inflammatory marker. This is also consistent with our findings that the L/A group with higher mortality rates had lower serum albumin levels. However, serum albumin levels can also be affected by a patient’s blood glucose, nutritional status, and liver function [13], so it also has certain limitations.

In critically ill patients with inflammation and tissue hypoxia, serum albumin and lactate levels show differences. Albumin and lactate each independently predict mortality, and the values inversely change by different mechanisms. Although plasma lactate and albumin are associated with cardiovascular diseases, previous studies demonstrated limited evidence about those systemic inflammatory biomarkers alone examining this reciprocal relationship to prognosis of cardiogenic shock. Consequently, more studies propose using the L/A ratio as a comprehensive indicator. This metric not only reflects oxygen supply and inflammation levels but also provides a more comprehensive representation of the patient’s current pathophysiological state through their combination, demonstrating advantages over single indicators. In recent years, applications of LAR as a combined biomarker have shown promising prognostic value across various clinical conditions. Research has demonstrated that LAR is an independent risk factor associated with poor prognosis in various medical conditions, including acute myocardial infarction, septic myocardial injury, intracerebral hemorrhage, heart failure, cardiac arrest survivors, severe sepsis, and septic shock [10], [23], [24], [25], [26]. Currently, no studies have been published that specifically address whether the LAR can be utilized to assess the prognosis of patients experiencing cardiogenic shock. Our study represents the first to evaluate the utility of the LAR in cardiogenic shock patients. The LAR combines lactate, a biomarker traditionally reflective of oxygen debt, with albumin, a biomarker that can represent acute inflammation. Both biomarkers often accompany critical illness to various degrees across a range of pathologies. Serum lactate production reflects oxygen debt secondary to the transition from aerobic to anaerobic metabolism; however, it is also influenced by stimulus of muscular Na-K ATPase, such as beta agonist exposure. Lactate clearance is additionally influenced by both renal and hepatic function. Inflammatory stimuli cause an acute decrease in serum albumin levels as a function of increased breakdown, decreased production, and changes in tissue distribution due to altered vascular permeability. Hypoalbuminemia may also exist in the setting of chronic malnutrition, chronic inflammatory states, and hepatic dysfunction. The dynamic relationship between pathologies underlying critical illness and their potential effect on lactate and albumin levels positions the LAR as a promising marker of worsening physiologic status, future development of cardiogenic shock, and/or mortality. Our study demonstrated that a higher LAR is associated with a poorer prognosis in patients suffering from cardiogenic shock, suggesting that this combined biomarker may be capable of being better indicative of the risk of cardiogenic shock patients. This association remained robust even when multivariate and subgroup analyses were conducted to mitigate the influence of confounding variables. Therefore, regarding the prognosis of individuals suffering from severe cardiogenic shock, a significant emphasis on the L/A ratio has important clinical implications.

A study conducted in Tabriz, Iran with a sample size of 151 individuals revealed that the LAR exhibited the highest AUC for predicting mortality in septic shock, suggesting its utility as a valuable prognostic factor [27]. Prior investigations have also highlighted the significance of elevated LAR levels upon admission as a strong predictor of in-hospital mortality in heart failure following myocardial infarction, outperforming lactate levels, SOFA score, and other relevant prognostic markers [28]. One previous study, derived from a prospective cohort conducted from September 2018 till February 2021 on adult patients presenting to the Emergency Department (ED) at a tertiary care center with sepsis or septic shock, has also confirmed that high LAR (≥0.115) on ICU admission is an independent risk factor for mortality in patients with sepsis or septic shock, which was slightly greater than our findings [29]. One retrospective cohort study with the data from the MIMIC-IV (v1.0) database has also confirmed that high LAR (≥1.1124) on ICU admission is an independent risk factor for 28-day mortality in patients with acute pancreatitis [30]. One retrospective analysis of the MIMIC-IV database has also confirmed that high LAR (>0.56) on ICU admission is an independent risk factor for 28-day mortality in ICU critical patients with coronary heart disease [31]. One retrospective analysis of the Medical Information Mart for Intensive Care III database has also confirmed that high LAR (>0.47) on ICU admission is an independent risk factor for 30-day mortality in ICU critical patients with acute myocardial infarction [10]. The LAR cutoff was higher than in our study. The target population of our study was shock patients, where lactate would be a little higher and albumin might be a little lower, so we had a higher LAR value. Nevertheless, there is currently a dearth of studies examining the correlation between LAR and cardiogenic shock patients. From a pathophysiological perspective, individuals with cardiogenic shock may exhibit heightened levels of tissue ischemia, cellular injury, and inflammatory reactions. These patients frequently endure more pronounced liver dysfunction and may additionally encounter multiple organ dysfunction syndrome (MODS). This group has received relatively less attention. To our knowledge, there have been no reports on the correlation between LAR levels and the prognosis of cardiogenic shock patients. Therefore, we conducted a retrospective study utilizing data from the The CardShock study database to investigate the association between LAR levels and the prognosis of 176 cardiogenic shock patients. Cox regression analyses revealed that a high LAR (>0.094) independently contributed to the risk of mortality at 90 days in cardiogenic shock patients. This discovery has the potential to assist clinicians in identifying patients at a higher risk, enabling them to intervene earlier and closely monitor their condition.

Several limitations of this study must be acknowledged. First, as a retrospective study, due to the selection/exclusion criteria established in the research, the analysis only included patients who obtained both lactate and albumin values within 3 h after shock recognition, which may introduce selection bias and result in samples that do not represent the target population. We adjusted for certain variables to enhance the accuracy of our results, we adjusted for certain variables to enhance the accuracy of our results. Second, the original dataset lacks key therapeutic variables such as albumin supplementation, mechanical circulatory support, or vasoactive agents use. These variables may simultaneously influence both LAR and prognostic treatment, thereby introducing residual confounding factors that constitute limitations of this study. Third, we focused solely on the initial LAR values recorded within 3 h of identifying cardiogenic shock, without tracking its subsequent dynamics; however, the initial LAR may provide a more precise prediction of the prognosis for cardiogenic shock. Lastly, this study is based on a single-center experience, and the limited number of enrolled patients may diminish the strength of our conclusions. Therefore, it would be advantageous to include patients from multiple centers worldwide to validate our findings, which we aim to pursue in future research. Despite these limitations, our investigation into the relationship between LAR and prognosis in patients with cardiogenic shock remains significant.

In conclusion, to the best of our knowledge, this study is the first to reveal that the LAR serves as an independent predictor of both 90-day and in-hospital mortality in ICU patients suffering from cardiogenic shock. The combined model form biomarkers had a higher-precision AUC, Sensitivity and Negative Predictive Value for predicting outcomes than models developed from single markers of lactate and albumin. Furthermore, our findings indicate a nonlinear relationship between LAR and 90-day mortality among these patients. Specifically, it was observed that survival rates decline as LAR increases in individuals experiencing cardiogenic shock. In addition, the LAR model shows optimal utility for intermediate-risk clinical decisions (30–75% threshold). Finally, these results suggest that LAR measured early in the course of cardiogenic shock may be a better discriminator of mortality and cardiogenic shock than either initial lactate or initial albumin alone.

## Supplementary Material

Supplementary Material

Supplementary Material
