# Supplementary material for: Lactate/albumin ratio predicts 90-day mortality of cardiogenic shock patients
Source: Open Med (Wars). 2026 Feb 20;21(1):20251355. doi: 10.1515/med-2025-1355 (PMC12919709; doi:10.1515/med-2025-1355)
Supplement: Supplementary file 1 — Supplementary Material [file j_med-2025-1355_suppl_001.docx]

**Table S1:** Missing data summary table

| **Variables** | **Number of missing values** | **Missing percent(%)** |
| --- | --- | --- |
| IABP-SHOCK II score | 58 | 32.95 |
| CardShock risk score | 9 | 5.11 |
| LVEF | 8 | 4.55 |
| diastolic BP | 8 | 4.55 |
| mean arterial pressure | 8 | 4.55 |
| BMI | 7 | 3.98 |
| systolic BP | 3 | 1.7 |
| NT-proBNP | 1 | 0.57 |
| CRP | 1 | 0.57 |
| **Characteristics** |  |  |
| Age | 0 | 0 |
| male | 0 | 0 |
| current smoker | 0 | 0 |
| ex-smoker | 0 | 0 |
| **Medical history** |  |  |
| coronary artery disease | 0 | 0 |
| Previous MI | 0 | 0 |
| Prior CABG | 0 | 0 |
| Previous MI or CABG | 0 | 0 |
| History of HFrEF | 0 | 0 |
| Diabetes mellitus | 0 | 0 |
| **Medications in use at admission** |  |  |
| ACEI | 0 | 0 |
| ARB | 0 | 0 |
| calcium-channel blockers | 0 | 0 |
| Beta-blocker | 0 | 0 |
| **Clinical presentation** |  |  |
| confusion | 0 | 0 |
| oliguria | 0 | 0 |
| ACS etiology | 0 | 0 |
| lung oedema on X-ray | 0 | 0 |
| vital status at 90 days | 0 | 0 |
| PCI complications | 0 | 0 |
| eGFR | 0 | 0 |
| **Laboratory test results at baseline** |  |  |
| haemoglobin | 0 | 0 |
| leucocytes | 0 | 0 |
| ALT | 0 | 0 |
| lactate | 0 | 0 |
| ALB | 0 | 0 |
| ALP | 0 | 0 |
| **Angiographic findings** |  |  |
| time from detection of shock to baseline | 0 | 0 |
| vital status at 90 days | 0 | 0 |

BMI = body mass index; CABG = coronary artery bypass grafting; MI=myocardial infarction; HFrEF = heart failure with reduced ejection fraction; ACEI = angiotensin-converting enzyme inhibitor; ARB = angiotensin receptor blocker; ACS = acute coronary syndrome; BP = blood pressure; LVEF = left ventricular ejection fraction; eGFR = estimated glomerular filtration rate; NT-proBNP = N-terminal prohormone of B-type natriuretic peptide; CRP = C-reactive protein; ALT = alanine aminotransferase; ALB=albumin; ALP=alkaline phosphatase; LAR=lactate/albumin ratio; PCI=Percutaneous Coronary Intervention.
