# Supplementary material for: Lactate/albumin ratio predicts 90-day mortality of cardiogenic shock patients
Source: Open Med (Wars). 2026 Feb 20;21(1):20251355. doi: 10.1515/med-2025-1355 (PMC12919709; doi:10.1515/med-2025-1355)
Supplement: Supplementary file 2 — Supplementary Material [file j_med-2025-1355_suppl_002.docx]

**Table S2:** collinearity analysis and eliminate the covariates with VIF> 5

|  | **VIF** |
| --- | --- |
| **Characteristics** |  |
| Age | 2.55 |
| Male | 1.94 |
| BMI | 2.13 |
| current smoker | 2.61 |
| ex-smoker | 2.06 |
| **Medical history** |  |
| coronary artery disease | 5.01 |
| Previous MI | 5.42 |
| Prior CABG | 2.10 |
| History of HFrEF | 2.41 |
| Diabetes mellitus | 1.95 |
| **Medications in use at admission** |  |
| ACEI | 2.13 |
| ARB | 1.83 |
| calcium-channel blockers | 1.55 |
| Beta-blocker | 2.91 |
| **Clinical presentation** |  |
| confusion | 1.47 |
| oliguria | 1.51 |
| ACS etiology | 1.60 |
| lung oedema on X-ray | 1.83 |
| mean arterial pressure | 1.60 |
| LVEF | 1.61 |
| eGFR | 2.40 |
| PCI complications | 1.90 |
| **Laboratory test results at baseline** |  |
| haemoglobin | 1.72 |
| leucocytes | 2.02 |
| NT-proBNP | 2.72 |
| CRP | 1.44 |
| ALT | 1.61 |
| ALP | 1.48 |
| **Angiographic findings** |  |
| time from detection of shock to baseline | 1.74 |

Since the VIF values for variables coronary artery disease and Previous MI were greater than 5, Previous MI was excluded from the final screening of covariates, while coronary artery disease was retained.
